# Clinical Impact of Pathogenic Variants in DNA Damage Repair Genes beyond *BRCA1* and *BRCA2* in Breast and Ovarian Cancer Patients

**DOI:** 10.3390/cancers14102426

**Published:** 2022-05-13

**Authors:** Whitney Espinel, Marjan Champine, Heather Hampel, Joanne Jeter, Kevin Sweet, Robert Pilarski, Rachel Pearlman, Kate Shane, Pamela Brock, Judith A. Westman, Lindsay Kipnis, Jilliane Sotelo, Anu Chittenden, Samantha Culver, Jill E. Stopfer, Katherine A. Schneider, Rosalba Sacca, Diane R. Koeller, Shraddha Gaonkar, Erica Vaccari, Sarah Kane, Scott T. Michalski, Shan Yang, Sarah M. Nielsen, Sara L. Bristow, Stephen E. Lincoln, Robert L. Nussbaum, Edward D. Esplin

**Affiliations:** 1 Huntsman Cancer Institute, Salt Lake City, UT 84112, USA; whitney.espinel@hci.utah.edu (W.E.); mjchampine@gmail.com (M.C.); 2 Ohio State University Medical Center, Columbus, OH 43210, USA; hhampel@coh.org (H.H.); joanne.jeter@hci.utah.edu (J.J.); kevin.sweet@osumc.edu (K.S.); rpilarski@ambrygen.com (R.P.); rachel.pearlman@osumc.edu (R.P.); kate.shane@osumc.edu (K.S.); pamela.brock@osumc.edu (P.B.); judith.westman@osumc.edu (J.A.W.); 3 Dana Farber Cancer Institute, Boston, MA 02215, USA; lindsay_kipnis@dfci.harvard.edu (L.K.); jilliane.sotelo@thermofisher.com (J.S.); anu_chittenden@dfci.harvard.edu (A.C.); samantha.culver@invitae.com (S.C.); jille_stopfer@dfci.harvard.edu (J.E.S.); katherine_schneider@dfci.harvard.edu (K.A.S.); saccar@mskcc.org (R.S.); dianer_koeller@dfci.harvard.edu (D.R.K.); shraddha.gaonkar@invitae.com (S.G.); erica.m.vaccari@gmail.com (E.V.); kanes@mskcc.org (S.K.); 4 Invitae, San Francisco, CA 94103, USA; scott.michalski@invitae.com (S.T.M.); yangshan88@gmail.com (S.Y.); sarah.nielsen@invitae.com (S.M.N.); sara.bristow@invitae.com (S.L.B.); steve.lincoln@me.com (S.E.L.); robert.nussbaum@invitae.com (R.L.N.)

**Keywords:** genetic testing, DNA damage repair, moderate-risk genes, clinical utility, breast cancer, ovarian cancer, clinical management

## Abstract

**Simple Summary:**

The clinical utility of positive findings in DNA damage-repair (DDR) genes *BRCA1* and *BRCA2* for the treatment of patients with breast or ovarian cancer is well established. However, multigene panel genetic testing for patients with breast and ovarian cancer now commonly includes DDR genes in addition to *BRCA1* and *BRCA2*, a number of which are considered moderate or low-risk genes. This study aimed to describe the clinical utility of positive results from genetic testing when the findings were in one of these other DDR genes. In a group of 101 women with positive findings in a cancer gene other than *BRCA1* or *BRCA2* (often in a DDR gene), nearly three-fifths (58%) had a clinical recommendation made based on their positive genetic test result and two-thirds (65%) had the clinician make recommendations for family members that may be at risk. This real-world data provides evidence that positive findings from genetic testing for moderate and low-risk genes, including DDR genes, can have clinical utility and can impact a patient’s clinical management.

**Abstract:**

Consensus guidelines for hereditary breast and ovarian cancer include management recommendations for pathogenic/likely pathogenic (P/LP) variants in *ATM*, *CHEK2*, *PALB2,* and other DNA damage repair (DDR) genes beyond *BRCA1* or *BRCA2*. We report on clinical management decisions across three academic medical centers resulting from P/LP findings in DDR genes in breast/ovarian cancer patients. Among 2184 patients, 156 (7.1%) carried a P/LP variant in a DDR gene. Clinical follow-up information was available for 101/156 (64.7%) patients. Genetic test result-based management recommendations were made for 57.8% (*n* = 59) of patients and for 64.7% (*n* = 66) of patients’ family members. Most recommendations were made for moderate-to-high risk genes and were consistent with guidelines. Sixty-six percent of patients (*n* = 39/59) implemented recommendations. This study suggests that P/LP variants in DDR genes beyond *BRCA1* and *BRCA2* can change clinical management recommendations for patients and their family members, facilitate identification of new at-risk carriers, and impact treatment decisions. Additional efforts are needed to improve the implementation rates of genetic-testing-based management recommendations for patients and their family members.

## 1. Introduction

The clinical utility of germline genetic testing for *BRCA1* and *BRCA2* has been established in patients with histories suggestive of hereditary breast and ovarian cancer [1,2]. However, mounting evidence has demonstrated that other DNA damage repair (DDR) genes, such as *ATM*, *CHEK2*, and *PALB2,* are associated with an increased breast, and potentially ovarian, cancer risk The risk associated with breast or ovarian cancer for each gene is variable and, for some, there may be no distinctive personal or family history characteristics that are predictive of carrying a pathogenic or likely pathogenic (P/LP) variant in one of these genes [3,4,5,6]. Furthermore, there is growing evidence for the role of an increasing number of DDR genes in cancer predisposition, prognosis, and predicting response to precision therapy [7]. As evidence has grown, demonstrating the association between P/LP variants in these DDR genes and breast and, perhaps ovarian cancer as well, these genes have been included on multi-gene panels that are becoming more frequently utilized in germline genetic testing for patients diagnosed with breast and/or ovarian cancer in the United States as well as globally [8,9,10]. A positive result in *ATM*, *CHEK2*, *PALB2,* or other DDR genes may result in modifications to interventions that clinicians consider when planning a patient’s clinical management and care. Such actionable interventions vary but could include clinical management changes based on consensus guidelines [11], implementation of Food and Drug Administration (FDA)-approved targeted therapies, or enrollment in clinical trials.

The real-world clinical impact of positive findings in *ATM*, *CHEK2*, *PALB2,* and other DDR genes conferring an increased risk of breast and/or ovarian cancer have not been well studied. As broad, multi-gene panel testing for these genes becomes more commonplace, it is critical to understand how clinicians counsel patients when positive findings are reported. In an effort to describe the real-world clinical impact of positive findings in *ATM*, *CHEK2*, *PALB2,* and other DDR genes in a cohort of breast and/or ovarian cancer patients, we describe whether provider-reported clinical recommendations changed, and which interventions were recommended based on genetic testing results. Furthermore, we explore whether the recommendations were implemented by the patient.

## 2. Materials & Methods

This study included a sample of patients with a personal history of breast and/or ovarian cancer who underwent clinician-ordered multi-gene panel germline genetic testing (Invitae, San Francisco, CA, USA) and received a P/LP variant in one of the ordered genes between 2014 and 2016 at three medical genetics clinics that are part of large academic health systems (Huntsman Cancer Institute, Salt Lake City, UT, USA; Ohio State University Medical Center, Columbus, OH, USA; and Dana Farber Cancer Institute, Boston, MA, USA).

The number and specific subset of genes tested in each individual on the previously validated platform [12] was chosen at the ordering clinicians’ discretion. Briefly, DNA extracted from blood or saliva samples were sequenced using a short-read next-generation sequencing (NGS) assay. A custom bioinformatics pipeline aligned sequencing reads and identified single nucleotide variants, small and large insertions or deletions, structural variants, and exon-level copy number variants (CNVs) [12,13,14]. Variants were analyzed and interpreted using the Sherloc framework, a points-based method that incorporates the joint consensus guidelines from the American College of Medical Genetics and Genomics and the Association for Molecular Pathology [15,16]. Based on the evidence, variants could be classified as benign or likely benign, uncertain significance, or P/LP.

For patients with a P/LP variant in *ATM*, *CHEK2*, *PALB2,* and other DDR genes (excluding *BRCA1* and *BRCA2*), ordering clinicians (comprised mostly of genetic counselors and some medical geneticists) completed a case-report form (CRF, Supplemental Methods). In addition to indicating breast and/or ovarian cancer (a requirement for study eligibility), clinicians reported on relevant clinical history, including a personal history of other cancers, a family history of breast or ovarian cancers, or a family history of other cancers. The CRF also collected information regarding clinical recommendations that had (or had not) changed based on the genetic testing results for either the patient or for the patient’s relatives. Based on patient medical records, clinicians indicated which, if any, recommendations patients completed (referred to as “implemented” in the CRF, see Supplement). In addition, clinicians were asked to indicate whether any implementation resulted in known clinical treatment interventions or outcomes and whether the testing result impacted their patient’s health outcome. Notably, information regarding the recommendations among family members was based on the presence of documentation in clinical notes in the proband’s medical records, but no demographic or other information was available for these family members.

All CRFs were sent to clinicians at the same time; therefore, data regarding implementation varied depending on the time between testing and CRF completion.

In addition to analyzing the real-world impact of these positive findings, an analysis of whether the positive findings were linked to actionable steps based on currently available interventions was investigated. In this analysis, actionable steps for each gene were defined as one of the three following groups: (1) management recommendations, including those derived from National Comprehensive Cancer Network (NCCN) guidelines [11,17,18,19,20,21,22,23,24]; (2) FDA-approved targeted therapies; and/or (3) clinical trial eligibility (Table S1). The cohort of patients with P/LP variants in *ATM*, *CHEK2*, *PALB2,* and other DDR genes (excluding *BRCA1* and *BRCA2*) were grouped based on the associated life-time risk of each gene in relation to the risk of breast and/or ovarian cancer relative to the general population lifetime incidence [25,26]: high (breast >40%, ovarian >10%), moderate (breast 20–39%, ovarian 5–10%), minimally increased (breast <20%, ovarian <5%), and related to risk for other cancers (established/preliminary risk for other cancer types but unclear breast and/or ovarian cancer risk) (Table S1) [3,6,27,28,29,30,31]. Patients with a P/LP variant in more than one gene were categorized according to the highest breast and/or ovarian cancer risk classification. Clinical recommendations and implementation for patients and their family were summarized by cancer risk. All analyses were summarized using descriptive statistics, using the Wilson method to calculate 95% confidence intervals (CIs).

## 3. Results

### 3.1. Patient Population

During the study period, 2184 patients with a personal history of breast and/or ovarian cancer were recommended for germline genetic testing per clinician assessment. Among them, 1932 (88.5%) did not have any P/LP variants in tested genes, 94 (4.3%) had P/LP variants in *BRCA1* or *BRCA2*, and 156 (7.1%) had P/LP variants in *ATM*, *CHEK2*, or another DDR gene other than *BRCA1* and *BRCA2*. CRFs were returned for 102 of 156 (65.4%) patients with P/LP variants in DDR genes other than *BRCA1* and *BRCA2*. One patient with follow-up information was male and was excluded from the analysis in order to investigate clinical management changes among a cohort of all females. Among the all-female cohort (*n* = 101), the majority were White (88.1%) and reported a family history of breast and/or ovarian cancer (62.7%) (Table 1). A personal history of breast cancer was most common (83.2%), followed by ovarian cancer (13.9%), with the remainder reporting a personal history of both breast and ovarian cancers. Median duration of follow-up between time of testing and time of CRF completion was 327 days (interquartile range, 397 days; range 63 to 1040 days).

**Figure 1:**
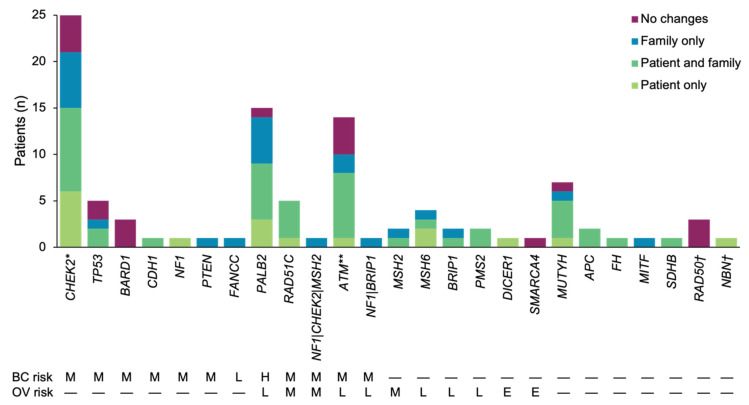
Distribution of P/LP variants in genes with known cancer risks and clinical recommendations, stratified by cancer risk. For each gene with P/LP variants, it was determined whether any results-based recommendations were made for the patient and/or their family members. Total number of patients with a P/LP variant in each gene is indicated. * One individual with a P/LP variant in *CHEK2* also had a P/LP variant in *MUTYH*. ** One individual with a P/LP variant in *ATM* also had a P/LP variant in *NBN*. ^†^*RAD50* and *NBN* do not have associated breast or ovarian cancer risks, but do have implications for clinical trial eligibility. BC, breast cancer; E, elevated but undefined risk due to limited data; H, high; L, minimally increased; M, moderate; OV, ovarian cancer; P/LP, pathogenic/likely pathogenic.

In total, P/LP variants in 23 genes were identified (Figure 1, Table S2). One third (*n* = 37, 36.6%) of patients had a P/LP variant in a gene associated with increased breast cancer risk, one third (*n* = 35, 34.7%) had a P/LP variant in a gene associated with an increase in both breast and ovarian cancer risks, and one eighth (*n* = 13, 12.9%) had a P/LP variant in a gene associated with increased ovarian cancer risk. The remaining patients had P/LP findings in genes associated with other HCS (*n* = 12, 11.9%) or no cancer risk (but with eligibility for clinical trials). The three most common genes with P/LP variants were *CHEK2*, *PALB2*, and *ATM*. Among genes with a moderate risk of breast cancer, most (83.3%, *n* = 5/6) had published management guidelines, with the remaining moderate-risk gene (*BARD1*) associated with potential eligibility for a clinical trial (Table S1). Three genes with high or moderate breast cancer risk and moderate or minimally increased ovarian cancer risk all had published management guidelines. The four genes with only a moderate or minimally increased risk of ovarian cancer also had published management guidelines.

### 3.2. Results-Based Management Recommendations for Patients

**Figure 2:**
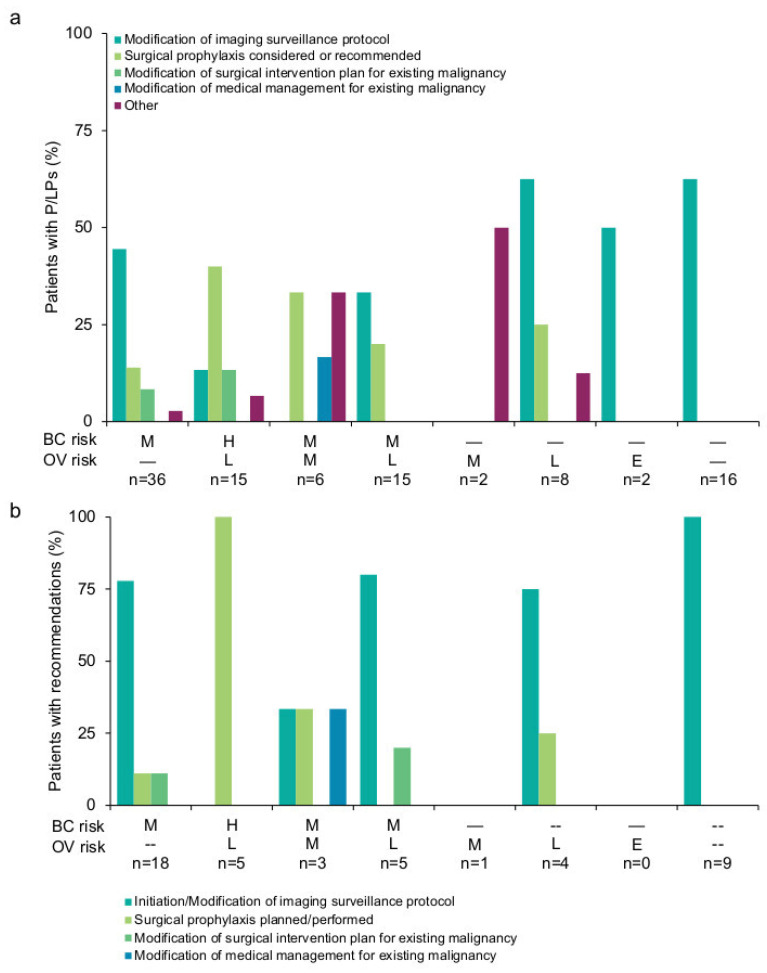
Clinical recommendations made to patients (**a**) and subsequent adherence (**b**). The percent of patients who received each recommendation was reported. The number of patients who received recommendations (numerator) versus the number of patients with a P/LP variant in each gene risk group (denominator) is reported in the *x*-axis. Modification of the medical management plan for existing malignancy included radiation therapy, chemotherapy, or other medications. Recommendations were made to patients with variants in the following genes: moderate breast cancer risk only (*CDH1*, *CHEK2*, *NF1*, and *TP53*); high breast cancer risk, minimally increased ovarian cancer risk (*PALB2*); moderate breast and ovarian cancer risks (*RAD51C*); moderate breast cancer risk, minimally increased ovarian cancer risk (*ATM*); moderate ovarian cancer risk only (*MSH2*); minimally increased ovarian cancer risk only (*BRIP1*, *MSH6*, *PMS2*); elevated but undefined ovarian cancer risk only (*DICER1*); and other cancer risk (*APC*, *FH*, *NBN*, *MUTYH*, *SDHB*). Other recommendations include: inclusion in a research protocol for PARP inhibitors, modification of colonoscopy schedule, screening for cancers other than existing malignancy, and discussion of pancreatic screening. Patients with P/LP variants in the following genes adhered to recommendations: moderate breast cancer only (*CDH1*, *CHEK2*, *NF1*, *TP53*); high breast cancer risk, minimally increased ovarian cancer risk (*PALB2*); moderate breast and ovarian cancer risks (*RAD51C*); moderate breast cancer risk, minimally increased ovarian cancer risk (*ATM*); moderate ovarian cancer risk only (*MSH2*); minimally increased ovarian cancer risk only (*BRIP1*, *MSH6*, *PMS2*); and other cancer risk (*APC*, *FH*, *MUTYH*, *NBN*, *SDHB*). BC, breast cancer; E, elevated but undefined risk due to limited data; H, high; L, minimally increased; M, moderate; OV, ovarian cancer; P/LP, pathogenic/likely pathogenic.

The specific recommendations were aggregated for the 59 (58.4%, 95% CI: 48.8–68.0%) patients for whom the clinician responded “Yes” to the following question: “For this patient, did positive genetic test results change clinical recommendations or counseling from those that would have been made, had genetic testing not been performed?” In total, 72 distinct management recommendations were made, the majority of which were made for P/LP variants in moderate-to-high risk genes for either breast or ovarian cancer (Figure 2a). Published management guidelines based on results were available for 49 (83.1%, 95% CI: 73.5–92.7%) patients. Among those with positive findings in a moderate-to-high-risk gene for either breast or ovarian cancer, the most commonly reported recommended intervention was the consideration or recommendation of surgical prophylaxis or modification of surgical intervention for the existing malignancy (*n* = 19), though there was no additional detail provided. Among patients with recommendations related to surgical plans, P/LP variants were detected in *PALB2* (*n* = 7), *TP53* (*n* = 2), *CDH1* (*n* = 1)—which have associated guidelines recommending discussion of risk-reducing mastectomy—and in *RAD51C* (*n* = 2), *BRIP1* (*n* = 1), and *MSH6* (*n* = 1)—which have associated guidelines for considering risk-reducing salpingo-oophorectomy. In addition, surgical recommendations were made for patients with P/LP variants in *CHEK2* (*n* = 3) and *ATM* (*n* = 2), even though these genes do not have any guidelines that recommend risk-reducing surgery, except as indicated by the patient’s family history. Modifications of imaging surveillance protocols were also common among patients. None of the clinicians indicated that chemoprevention was recommended for the patients. One patient with a P/LP variant in *RAD51C* was included in a poly ADP-ribose polymerase inhibitor (PARPi) clinical trial that required documentation of a P/LP variant in a homologous recombination deficiency (HRD) gene for enrollment.

Clinicians reported that 39 (39/59, 66.1%, 95% CI: 54.0–78.2%) patients adhered to at least one recommendation (CRF question: “For this patient, were recommendations implemented based on genetic test results?”). In total, 51 recommendations were implemented (Figure 2b). Cancer risk groups with the highest adherence rates included moderate ovarian cancer risk only (*n* = 1/1, 100%), other cancer risk (*n* = 9/10, 90.0%), minimally increased ovarian cancer risk only (*n* = 5/6, 83.3%), moderate breast cancer risk only (*n* = 13/19, 68.4%), and high breast/minimally increased ovarian cancer risk (*n* = 6/9, 66.7%). Patients with P/LP variants in genes with moderate breast cancer risk and moderate or minimally increased ovarian cancer risk had lower adherence rates (*n* = 2/5, 40.4%; *n* = 3/8, 37.5%, respectively). No patients with P/LP variants associated with an elevated but undefined ovarian cancer risk adhered to recommendations. The initiation or modification of imaging surveillance protocols were the most common recommendation adhered to, followed by surgical prophylaxis.

Though limited in availability, positive outcomes were reported for a number of patients (CRF question: “Did the implementation[s] based on genetic test for your patient, and/or their family members, result in any known clinical outcomes?”) in whom responses were limited to only those patients where the patient adhered to a results-based recommendation. Three patients with P/LP variants in genes with a moderate risk of breast cancer were disease free following prophylactic surgery or a modification of the surgical plan for an existing malignancy. An additional three patients with a P/LP in *PALB2* (high breast cancer and elevated, albeit low, ovarian cancer risks) were disease free after prophylactic surgery (*n* = 2) or modification of medical management for an existing malignancy (*n* = 1). Furthermore, among patients adhering to at least one recommendation, the reporting clinician indicated that the genetic test result impacted the patient’s health outcome for 24 patients with a P/LP variant in *ATM*, *BRIP1*, *CHEK2*, *MSH2*, *MSH6*, *PALB2*, *PMS2*, *RAD51C*, *TP53*. No clinician answered “No” to the CRF question, “Did the genetic test result impact the patient’s health outcome?”, for any patients meeting these criteria; this information was not available (“Unknown”) for six patients (*CDH1*, *CHEK2*, *MSH6*, *NF1*, *PALB2*).

Among the 42 patients with no changes in clinical recommendations, nearly three-quarters (73.8%) had positive findings in genes with a moderate-to-high risk of breast and/or ovarian cancer. Clinicians reported reasons for a lack of recommendations in 35 cases. The most common reasons included advanced/metastatic cancer or death prior to receiving genetic testing results (37.1%) or a lack of clear recommendations that could be made based on the identified variant (e.g., low-penetrance variants or, possibly, mosaic findings) (25.7%) (Table 2). In four cases, patients did not have clinical management changes because their management had already been changed due to their family or personal history of cancer prior to genetic testing, and the positive findings did not modify management already implemented based on family or personal history.

### 3.3. Results-Based Management Recommendations for Family Members

**Figure 3:**
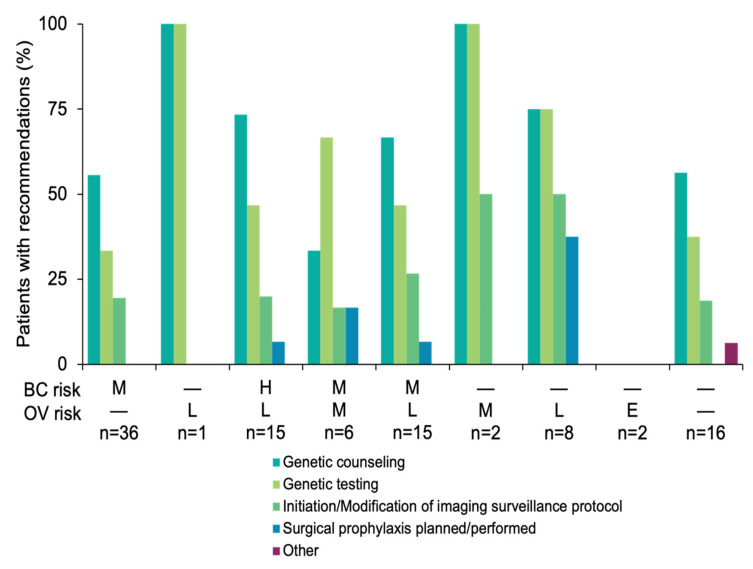
Clinical recommendations made to family members. The percent of family members who received each recommendation was reported. The number of patients for whom family members were reported to receive recommendations (numerator) versus the number of patients with a P/LP variant in each gene risk group (denominator) is reported in the *x*-axis. Recommendations were made to family members of patients with variants in the following genes: moderate breast cancer risk only (*CDH1*, *CHEK2*, *PTEN*, *TP53*); minimally increased breast cancer risk only (*FANCC*); moderate breast cancer and minimally increased ovarian cancer risk (*PALB2*); moderate breast and ovarian cancer risks (*NF1*, *RAD51C*); moderate breast cancer risk and minimally increased ovarian cancer risk (*ATM, NF1|BRIP1*); moderate ovarian cancer risk only (*MSH2*); minimally increased ovarian cancer risk only (*BRIP1*, *MSH6*, *PMS2*); other cancer risks (*APC*, *FH*, *MITF*, *MUTYH*, *SDHB*). Other recommendations include: carrier screening. Other family member implementation response: colonoscopy and family in process of receiving genetic testing. BC, breast cancer; E, elevated but undefined risk due to limited data; H, high; L, minimally increased; M, moderate; OV, ovarian cancer; P/LP, pathogenic/likely pathogenic.

Clinicians made 141 different results-based recommendations regarding patients’ family members in 65 cases (64.4%, 95% CI: 55.1–73.7%). Genetic counseling and/or testing was recommended most often (105/141, 74.5%) (Figure 3). Adherence to recommendations were not analyzed as this was collected via clinical notes in the patients’ medical records.

## 4. Discussion

This study demonstrates that, among patients with breast or ovarian cancer, positive genetic test results in *ATM, CHEK2, PALB2,* and other DDR genes impact clinical recommendations for the majority of patients and/or their family members. When no results-based recommendations were made, it was most commonly because the patient was diagnosed with advanced cancer and no further recommendations were deemed appropriate. It is possible that undergoing genetic testing earlier in these patients may have resulted in an improved outcome for the patient via precision therapy, earlier second primary cancer detection, or prevention. Results-based recommendations were consistent with current established guidelines [11]. For example, chemoprevention was not recommended, as expected from recent published studies demonstrating that the proportion of eligible patients pursuing chemoprevention remains low in actual practice [32,33,34,35]. In addition, results-based recommendations were more frequently implemented by patients with a positive finding in high-risk genes compared with the genes in other risk groups. However, patient adherence rates across all patients and their relatives indicate that identifying P/LP variants in *ATM, CHEK2, PALB2,* and other DDR genes associated with increased risk of breast and ovarian cancer also impact patient care.

While clinical trial eligibility was not a pre-specified choice in this survey, one clinician reported discussing the topic with a patient. Additional patients may have been informed of this option but not reported. Though this is anecdotal and not an anticipated finding from this study, it is certainly of interest. Such discussions could provide opportunities for patients with limited treatment options to consider enrollment in clinical treatment trials that are tailored to their personal genetic test results, consistent with NCCN guidelines stating the “NCCN believes that the best management of any patient with cancer is in a clinical trial.” Further studies examining whether clinical trials are routinely discussed as an option for patients may provide more insights into the barriers and facilitators of identifying eligible patients.

This was an observational study of clinical utility and implementation and therefore has limitations, including the retrospective design with a small sample size and inclusion criteria and interpretation of CRF questions (e.g., definition of implementation meaning taking the logistical steps needed to facilitate patient adherence vs. the patient following through with the recommendation) that were dependent on testing practices of different providers. The CRF was designed to collect real-world data in a comprehensive and consistent manner, resulting in questions that were multiple choice, with an option to provide free-text responses for more detail. The CRF did not go into details of the specifics of each way that clinical recommendations could have changed (e.g., when surgical prophylaxis was considered or recommended, what was the specific surgical plan?). In addition, the CRF did not collect information about the stage of breast or ovarian cancer or the prior treatment regimens that had been recommended and implemented for each patient. While this information would be invaluable to understanding the real-world implications of positive findings in *ATM, CHEK2, PALB2,* and other DDR genes, this had to be balanced with the time to complete the CRF. These data suggest future studies analyzing additional aspects of clinical care are warranted. The ordering clinicians were genetic counselors and medical geneticists who were part of medical genetics clinics that are part of large academic health care systems and benefit from a robust integrated electronic health record for patients. However, in a number of cases, patients and/or their families were lost to follow-up or information regarding clinical recommendations may not have been accessible to the ordering clinician completing the survey (e.g., the genetic test was a part of a referral; consultation was obtained adjacent to the treating clinician at a different hospital or clinic; documentation in the patient’s medical record was limited). Another consideration related to the ordering providers is that there may have been bias in the types of recommendations and subsequent patient uptake (i.e., recommending family testing vs. other treatments) that were reported due to the types of clinicians completing the CRFs. Future studies including additional types of specialty clinicians will help clarify the decision-making process following genetic testing results. A final point to consider is that the cancer-risk associations were based on the current literature and applied to recommendations that were made more than 5 years ago. Thus, while it may appear that some of the gene-specific recommendations might be deemed inappropriate according to current understanding of gene–disease relationships, the recommendations were justified by guidelines at the time of genetic testing. For example, *NBN* and *RAD50* were once considered to be associated with increased breast/ovarian cancer risk [36,37], but are no longer thought to be associated with cancer risk [30,31]. However, findings in these genes do make cancer patients eligible for clinical treatment trials (*NBN*, NCT02401347, NCT04171700; *RAD50*, NCT02401347, NCT02286687).

## 5. Conclusions

These data suggest there is real-world clinical utility in genetic testing that includes DDR genes, other than *BRCA1* and *BRCA2,* that have known associations with hereditary breast, ovarian, and other cancers. These findings align with clinical management guidelines that have expanded to include these genes. For example, the American Society of Breast Surgeons recently recommended that any patient with a personal history of breast cancer should receive genetic testing with a gene panel containing *ATM*, *CHEK2*, *PALB2,* and other DDR genes that are known to be associated with an increased risk of breast and ovarian cancer [38]. Additional studies of how such findings impact real-world clinical care will continue to elucidate the benefit of genetic testing in precision therapy, clinical management, and shortened time to diagnosis.

## Figures and Tables

**Table 1:** Demographics and clinical characteristics of 101 female patients with P/LP variants in genes beyond *BRCA1* and *BRCA2*.

Characteristic, *n* (%)	Patients (*n* = 101)
Ethnicity ^a^	
White/Caucasian	89 (88.1)
Black/African American	4 (4.0)
Ashkenazi Jewish	3 (3.0)
Other	4 (4.0)
Unknown	1 (1.0)
Age at testing ^a^	
20–29 years	3 (3.0)
30–39 years	5 (5.0)
40–49 years	25 (24.8)
50–59 years	31 (30.7)
60–69 years	27 (26.7)
70–79 years	6 (5.9)
≥80 years	4 (4.0)
Personal Br/Ov Ca history ^a,b^	
Breast cancer	84 (83.2)
Ovarian cancer	14 (13.9)
Breast and ovarian cancers	3 (3.0)
Other personal cancer history (in addition to Br/Ov Ca)	
Yes	19 (18.8)
No	77 (76.2)
Not reported	5 (5.0)
Family Br/Ov Ca history ^a^	
Breast cancer	44 (43.6)
Ovarian cancer	4 (4.0)
Breast and ovarian cancers	15 (14.9)
No	17 (16.8)
Not reported	22 (21.8)
Family non-Br/Ov Ca history	
Yes	62 (60.4)
No	38 (37.6)
Not reported	2 (2.0)
Previous genetic testing ^a^	
Patient only	4 (4.0)
Patient and relative(s)	2 (2.0)
Relative(s) only	2 (2.0)
None	93 (91.1)
Number of genes on ordered test	
1–5 genes	4 (4.0)
6–15 genes	16 (15.8)
16–50 genes	76 (75.2)
51+ genes	5 (5.0)

^a^ Percentages do not sum to 100% due to rounding. ^b^ All included patients had a personal history of breast and/or ovarian cancer. Br/Ov Ca, breast/ovarian cancer; P/LP, pathogenic/likely pathogenic.

**Table 2:** Reasons for not making results-based recommendations (*n* = 43).

Reason for No Results-Based Recommendations	Number (Percentage) of Patients
Patient already had advanced/metastatic cancer (or died before results available)	13 (30.2)
Gene/Variant/Finding with no clear recommendations available ^a^	9 (20.9)
Patient had previous interventions before genetic testing due to personal or family history of cancer	4 (9.3)
Recommendations for patient’s current cancer diagnosis superseded recommendations for genetic testing result or no further recommendations were applicable at this point in time given the patient’s current cancer diagnosis	6 (14.0)
Patient preference: patient did not follow recommendations or followed alternative recommendations	3 (7.0)
No information provided	8 (18.6)

^a^ These include: *BARD1* (*n* = 1), *CHEK2* (*n* = 2), *MITF* (*n* = 1), *MUTYH* (*n* = 1), *RAD50* (*n* = 1), and *TP53* mosaic variant (*n* = 3). While there may not have been clear guidelines due to the variant type, all genes included in testing were clinically actionable in some way (clinical management guidelines, FDA-approved treatments, and/or clinical trial eligibility [see Table S1]).

## Data Availability

The data that support the findings of this study are available from the corresponding author upon reasonable request.

## Supplementary Materials

The following supporting information can be downloaded at: https://www.mdpi.com/article/10.3390/cancers14102426/s1, Supplemental Methods: Case report forms completed by clinicians for each patient; Table S1: Summary of clinically actionable steps by gene; Table S2: Summary of genetic findings. References [11,17,18,19,20,21,22,23,24] are cited in the Supplementary Materials.
