# Supplementary material for: Clinical Impact of Pathogenic Variants in DNA Damage Repair Genes beyond BRCA1 and BRCA2 in Breast and Ovarian Cancer Patients
Source: Cancers (Basel). 2022 May 13;14(10):2426. doi: 10.3390/cancers14102426 (PMC9139211; doi:10.3390/cancers14102426)
Supplement: Supplementary file 1 [file cancers-14-02426-s001.zip › cancers-1700686-supplementary.pdf]

SUPPLEMENT

Supplement to “Impact of finding pathogenic variants beyond *BRCA1* and *BRCA2* on management of breast and ovarian cancer patients”

Authors: Whitney Espinel, Marjan Champine, Heather Hampel, Joanne Jeter, Kevin Sweet, Robert Pilarski, Rachel Pearlman, Kate Shane Pamela Brock, Judith A. Westman, Lindsay Kipnis, Jilliane Sotelo, Anu Chittenden, Samantha Culver, Jill Stopfer, Katherine Schneider, Rosalba Sacca, Diane Koeller, Shraddha Gaonkar, Erica Vaccari, Sarah Kane, Scott T. Michalski, Shan Yang, Sarah Nielsen, Sara L. Bristow, Stephen E. Lincoln, Robert L. Nussbaum, Edward D. Esplin

Supplemental Methods: Case report forms completed by clinicians for each patient
Table S1: Summary of clinically actionable steps by gene
Table S2: Summary of genetic findings

Supplemental Methods

non-BRCA Clinical Utility study

Start of Block: Default Question Block

Q1 Thank you for collaborating in this study and sharing your clinical experiences. As you know, through your input we are aiming to catalog the clinical impact of positive genetic test results (i.e. likely pathogenic/pathogenic variants) in cancer predisposition genes, excluding BRCA 1/2, in patients undergoing genetic testing for suspected HBOC or a related syndrome.

For each identified patient, using a short series of questions, we aim to capture brief summary details of the clinical management decisions made for each patient. We understand that information may not be available for some patients. By completing this survey you consent to the de-identified responses being analyzed and presented or published, as appropriate. This study is authorized under a protocol approved by Western IRB and Invitae will keep all clinician-provided data in its secure, HIPAA-compliant environment.

Q2 For this patient, did positive genetic test results change clinical recommendations or counseling from those that would have been made, had genetic testing not been performed?

- ○ Yes (1)
- ○ No (2)
- ○ Unknown (3)
- ○ Other, please explain (4) ____

Display This Question:

If For this patient, did positive genetic test results change clinical recommendations or counseling... = Yes

Q3 If yes, what clinical recommendations or counseling did positive genetic test results change? (please select all that apply)

- ☐ modification of imaging surveillance protocol (1)
- ☐ consideration of surgical prophylaxis (2)
- ☐ surgical prophylaxis recommended (3)
- ☐ modification of surgical intervention plan for existing malignancy (4)
- ☐ modification of medical management plan for existing malignancy (radiation therapy, chemotherapy, other medications) (5)
- ☐ discontinuation of current surveillance protocol based on test result (6)
- ☐ other, please explain (7) ____

Q4 For this patient's family members, did positive genetic test results in the patient change clinical recommendations or counseling from those that would have been made for the family members, had genetic testing not been performed?

- ○ Yes (1)
- ○ No (2)
- ○ Unknown (3)
- ○ Other, please explain (4) ____

Display This Question:

If For this patient's family members, did positive genetic test results in the patient change clinic... = Yes

Q5 If yes, what clinical recommendations or counseling for the patient's family members did positive genetic test results change? (please select all that apply)

- ☐ recommendation to see healthcare provider for genetic counseling (1)
- ☐ genetic testing (2)
- ☐ initiation of imaging surveillance protocol (3)
- ☐ modification of imaging surveillance protocol (4)
- ☐ consideration of surgical prophylaxis (5)
- ☐ surgical prophylaxis recommended (6)
- ☐ modification of surgical intervention plan for existing malignancy (7)
- ☐ modification of medical management plan for existing malignancy (radiation therapy, chemotherapy, other medications) (8)
- ☐ discontinuation of current surveillance protocol based on a genetic test result (9)
- ☐ Other, please explain (10) ____

Q6 For this patient, were recommendations implemented based on genetic test results?

- ○ Yes (1)
- ○ No (2)
- ○ Unknown (3)
- ○ Other, please explain (4) ____

Display This Question:

If For this patient, were recommendations implemented based on genetic test results? = Yes

Q7 If yes, what was implemented? (please select all that apply)

- ▢ initiation of imaging surveillance protocol (1)
- ▢ modification of imaging surveillance protocol (2)
- ▢ surgical prophylaxis planned or performed (3)
- ▢ modification of surgical intervention for existing malignancy (4)
- ▢ modification of medical management for existing malignancy (radiation therapy, chemotherapy, other medications) (5)
- ▢ chemopreventive medication initiated or planned (6)
- ▢ Unknown (7)
- ▢ Other, please explain (8) ____

Display This Question:

If For this patient, were recommendations implemented based on genetic test results? = No

Q16 If not, why? (please select all that apply)

- ▢ patient preference (1)
- ▢ lack of insurance coverage (2)
- ▢ lost to follow up (3)
- ▢ followed alternative recommendations (4)
- ▢ Unknown (5)
- ▢ Other, please explain (6) ____

Q8 For this patient's family members, were recommendations implemented based on genetic test results (based on positive or negative test results)?

- ○ Yes (1)
- ○ No (2)
- ○ Unknown (3)
- ○ Other, please explain (4) ____

Display This Question:

If For this patient's family members, were recommendations implemented based on genetic test results... = Yes

Q10 If yes, what was implemented? (please select all that apply)

- ▢ genetic testing (1)
- ▢ initiation of imaging surveillance protocol (2)
- ▢ modification of imaging surveillance protocol (3)
- ▢ surgical prophylaxis planned or performed (4)
- ▢ modification of surgical intervention for existing malignancy (5)
- ▢ modification of medical management for existing malignancy (radiation therapy, chemotherapy, other medications) (6)
- ▢ chemopreventive medication initiated or planned (7)
- ▢ Other, please explain (8) ____

Display This Question:

If For this patient's family members, were recommendations implemented based on genetic test results... = No

Q11 If not, why? (please select all that apply)

- ☐ patient preference (1)
- ☐ lack of insurance coverage (2)
- ☐ lost to follow up (3)
- ☐ followed alternative recommendations (4)
- ☐ Unknown (5)
- ☐ Other, please explain (6) ____

Q12 Did the implementation(s) based on genetic test for your patient, and/or their family members, result in any known clinical outcomes? (Please select all that apply)

- ☐ genetic testing identified an additional family member(s) as carrier(s) (5)
- ☐ patient is disease free after receiving modified medical management for existing malignancy (radiation therapy, chemotherapy, other medications) (6)
- ☐ new cancer diagnosed due to genetic testing based surveillance (7)
- ☐ patient is disease free after undergoing modified surgical intervention for malignancy (8)
- ☐ biopsy resulting in benign findings (9)
- ☐ patient is disease free after undergoing prophylactic surgical intervention (10)
- ☐ treatment related complication (11)
- ☐ No (2)
- ☐ Unknown (3)
- ☐ Other, please explain (4) ____

Q14 In your opinion, did the genetic test result impact the patient's health outcome?

○ Yes (1)

○ No (2)

○ Unknown (3)

○ If yes, please explain (4) ____

End of Block: Default Question Block

Table S1. Summary of clinically actionable steps by gene

	Cancer Risk					
Gene	Breast cancer	Ovarian cancer	Other cancer	Management Guidelines	FDA-approved cancer treatment	Cancer-gene specific clinical treatment trials
BARD1	Moderate	—	—	No	No	Yes
CDH1	Moderate	—	Yes	Yes (NCCN BOP1)	No	No
CHEK2	Moderate	—	—	Yes (NCCN BOP1; CRC2)	No	Yes
NF1	Moderate	—	Yes	Yes (NCCN BOP1)	No	No
PTEN	Moderate	—	Yes	Yes (NCCN BOP1)	No	Yes
TP53	Moderate	—	—	Yes (NCCN BOP1)	No	Yes
FANCC	Low	—	—	No	No	Yes
PALB2	High	Low	Yes	Yes (NCCN BOP1)	No	Yes
RAD51C	Moderate	Moderate	—	Yes (NCCN BOP1)	No	Yes
ATM	Moderate	Low	Yes	Yes (NCCN BOP1)	No	Yes
MSH2	—	Moderate	Yes	Yes (NCCN BOP1; CRC2)	Yes	No
BRIP1	—	Low	—	Yes (NCCN BOP1)	No	Yes
MSH6	—	Low	Yes	Yes (NCCN BOP1; CRC2)	Yes	No
PMS2	—	Low	Yes	Yes (NCCN BOP1; CRC2)	Yes	No
DICER1	—	Elevated[a]	Yes	Yes (Other3)	No	No
SMARCA4	—	Elevated[a]	—	No	No	Yes
APC	—	—	Yes	Yes (NCCN CRC2)	No	No
FH	—	—	Yes	Yes (Other[4,5])	No	No
MITF	—	—	Yes	No	No	No
MUTYH	—	—	Yes	Yes (NCCN CRC2)	No	No
NBN[b]	—	—	—	Yes (NCCN BOP1)	No	Yes

RAD50[b]	—	—	—	No	No	Yes
SDHB	—	—	Yes	Yes (Other[6–9])	No	No

BOP, breast, ovarian, prostate cancer; CRC, colorectal cancer; MI, minimally increased; NCCN; National Comprehensive Cancer Network.

Table S2. Summary of genetic findings[a]

Gene	hgvsP	Protein change	Interpretation	Count
APC	NM_000038.5:c.70C>T	p.Arg24*	pathogenic	2
ATM	Deletion (exons 2-63)	exons 2-63	pathogenic	1
ATM	NM_000051.3:c.1290_1291delTG	p.Cys430*	pathogenic	1
ATM	NM_000051.3:c.1561_1562delAG	p.Glu522IlefsX43	pathogenic	1
ATM	NM_000051.3:c.1564_1565delGA	p.Glu522Ilefs*43	pathogenic	1
ATM	NM_000051.3:c.1915_1916insT	p.Asp639Valfs*2	pathogenic	1
ATM	NM_000051.3:c.2251-10T>G	p.?	pathogenic	1
ATM	NM_000051.3:c.3450_3454delAAAAT	p.Arg1150Serfs*14	pathogenic	1
ATM	NM_000051.3:c.3802delG	p.Val1268*	pathogenic	1
ATM	NM_000051.3:c.5290delC	p.Leu1764Tyrfs*12	pathogenic	1
ATM	NM_000051.3:c.6397C>T	p.Gln2133*	pathogenic	1
ATM	NM_000051.3:c.6404_6405insTT	p.Arg2136*	pathogenic	1
ATM	NM_000051.3:c.7271T>G	p.Val2424Gly	pathogenic	3
BARD1	NM_000465.3:c.1325delC	p.Pro442Leufs*33	pathogenic	1
BARD1	NM_000465.3:c.1935_1954dupTGAACAGGAAGAAAAGTATG	p.Glu652Valfs*69	likely_pathogenic	1
BARD1	NM_000465.3:c.745dupA	p.Ile249Asnfs*9	pathogenic	1
BRIP1	NM_032043.2:c.1629-1G>T	p.?	likely_pathogenic	1
BRIP1	NM_032043.2:c.1871C>A	p.Ser624*	pathogenic	1
BRIP1	NM_032043.2:c.2255_2256delAA	p.Lys752Argfs*12	pathogenic	1
CDH1	NM_004360.3:c.1565+2dupT	p.?	likely_pathogenic	1
CHEK2	NM_007194.3:c.1100delC	p.Thr367Metfs*15	pathogenic	9
CHEK2	NM_007194.3:c.1283C>T	p.Ser428Phe	pathogenic_low_penetrance	2
CHEK2	NM_007194.3:c.277delT	p.Trp93GlyfsX17	pathogenic	1
CHEK2	NM_007194.3:c.349A>G	p.Arg117Gly	likely_pathogenic	1
CHEK2	NM_007194.3:c.433C>T	p.Arg145Trp	likely_pathogenic	1
CHEK2	NM_007194.3:c.470T>C	p.Ile157Thr	pathogenic_low_penetrance	8
CHEK2	NM_007194.3:c.683+1G>T	p.?	likely_pathogenic	1
CHEK2	NM_007194.3:c.909-?_1095+?del	null	pathogenic	3
DICER1	NM_177438.2:c.535delC	p.Ala180Glnfs*3	pathogenic	1
FANCC	NM_000136.2:c.996+1G>T	p.?	likely_pathogenic	1
FH	NM_000143.3:c.1431_1433dupAAA	p.Lys477dup	likely_pathogenic	1

MITF	NM_000248.3:c.952G>A	p.Glu318Lys	pathogenic	1
MSH2	NM_000251.2:c.1277-?_1386+?del	null	pathogenic	1
MSH2	NM_000251.2:c.2089T>C	p.Cys697Arg	pathogenic	1
MSH2	NM_000251.2:c.942+3A>T	p.?	pathogenic	1
MSH6	NM_000179.2:c.2535dupT	p.Glu846*	pathogenic	1
MSH6	NM_000179.2:c.3238_3239delCT	p.Leu1080Valfs*12	pathogenic	1
MSH6	NM_000179.2:c.3261dupC	p.Phe1088Leufs*5	pathogenic	1
MSH6	NM_000179.2:c.3850_3857dupACGTTCCT	p.Tyr1287Argfs*43	pathogenic	1
MUTYH	NM_001128425.1:c.1187G>A	p.Gly396Asp	pathogenic	7
MUTYH	NM_001128425.1:c.1438G>T	p.Glu480*	pathogenic	1
NBN	NM_002485.4:c.657_661delACAAA	p.Lys219Asnfs*16	pathogenic	1
NBN	NM_002485.4:c.698_701delAACA	p.Lys233Serfs*5	pathogenic	1
NF1	NM_000267.3:c.-383-?_60+?del	null	pathogenic	1
NF1	NM_000267.3:c.2033dupC	p.Ile679Aspfs*21	pathogenic	1
NF1	NM_000267.3:c.2709G>A	p.=	likely_pathogenic	1
PALB2	NM_024675.3:c.172_175delTTGT	p.Gln60Argfs*7	pathogenic	1
PALB2	NM_024675.3:c.212-2A>G	p.?	likely_pathogenic	1
PALB2	NM_024675.3:c.2386G>T	p.Gly796X	pathogenic	1
PALB2	NM_024675.3:c.2920_2921delAA	p.Lys974Glufs*5	pathogenic	1
PALB2	NM_024675.3:c.2T>C	p.Met1?	likely_pathogenic	1
PALB2	NM_024675.3:c.3026delC	p.Pro1009LeufsX6	pathogenic	1
PALB2	NM_024675.3:c.3113G>A	p.Trp1038*	pathogenic	2
PALB2	NM_024675.3:c.3323delA	p.Tyr1108SerfsX16	pathogenic	1
PALB2	NM_024675.3:c.3351-?_*297+?del}	null	pathogenic	1
PALB2	NM_024675.3:c.3549C>A	p.Tyr1183*	pathogenic	1
PALB2	NM_024675.3:c.3549C>G	p.Tyr1183*	pathogenic	1
PALB2	NM_024675.3:c.509_510delGA	p.Arg170Ilefs*14	pathogenic	1
PALB2	NM_024675.3:c.757_758delCT	p.Leu253IlefsX3	pathogenic	1
PALB2	NM_024675.3:c.758dupT	p.Ser254Ilefs*3	pathogenic	1
PMS2	Deletion (Exon 14)	Exon 14	pathogenic	2
PTEN	NM_000314.4:c.389G>A	p.Arg130Gln	pathogenic	1
RAD50	NM_005732.3:c.2202delC	p.Met735*	pathogenic	1

RAD50	NM_005732.3:c.2707delA	p.Arg903Glufs*3	pathogenic	1
RAD50	NM_005732.3:c.326_329delCAGA	p.Thr109Asnfs*20	pathogenic	1
RAD51C	NM_058216.2:c.224dupA	p.Tyr75*	pathogenic	1
RAD51C	NM_058216.2:c.577C>T	p.Arg193*	pathogenic	1
RAD51C	NM_058216.2:c.701C>G	p.Ser234*	pathogenic	1
RAD51C	NM_058216.2:c.93delG	p.Phe32Serfs*8	pathogenic	2
SDHB	NM_003000.2:c.296G>A	p.Gly99Asp	likely_pathogenic	1
SMARCA4	NM_001128849.1:c.2439-?_3546+?del	null	pathogenic	1
TP53	NM_000546.5:c.-202-?_*1207+?del}	null	pathogenic	1
TP53	NM_000546.5:c.542G>A	p.Arg181His	pathogenic	1
TP53	NM_000546.5:c.584T>C	p.Ile195Thr	pathogenic	1
TP53	NM_000546.5:c.742C>T	p.Arg248Trp	pathogenic	1
TP53	NM_000546.5:c.790delC	p.Leu264Tyrfs*81	pathogenic	1

[a]Patients with more than one pathogenic or likely pathogenic variant are represented more than once.

References

1. National Comprehensive Cancer Network. Genetic/Familial high-risk assessment: breast, ovarian and pancreatic (version 1.2020). https://www.nccn.org/professionals/physician_gls/pdf/genetics_bop.pdf. Published December 4, 2019. Accessed March 27, 2020.

2. National Comprehensive Cancer Network. Genetic/Familial High-Risk Assessment: Colorectal (version 3.2019). https://www.nccn.org/professionals/physician_gls/pdf/genetics_colon.pdf. Published December 13, 2019. Accessed March 27, 2020.

3. Schultz KAP, Williams GM, Kamihara J, et al. DICER1 and Associated Conditions: Identification of At-risk Individuals and Recommended Surveillance Strategies. *Clin Cancer Res*. 2018;24(10):2251-2261.

4. Menko FH, Maher ER, Schmidt LS, et al. Hereditary leiomyomatosis and renal cell cancer (HLRCC): renal cancer risk, surveillance and treatment. *Fam Cancer*. 2014;13(4):637-644.

5. Pithukpakorn M, Toro JR. Hereditary Leiomyomatosis and Renal Cell Cancer. In: Adam MP, Ardinger HH, Pagon RA, et al., eds. *GeneReviews®*. Seattle (WA): University of Washington, Seattle; 2006.

6. Lenders JWM, Duh Q-Y, Eisenhofer G, et al. Pheochromocytoma and paraganglioma: an endocrine society clinical practice guideline. *J Clin Endocrinol Metab*. 2014;99(6):1915-1942.

7. Lefebvre M, Foulkes WD. Pheochromocytoma and paraganglioma syndromes: genetics and management update. *Curr Oncol*. 2014;21(1):e8-e17.

8. Else T, Greenberg S, Fishbein L. Hereditary Paraganglioma-Pheochromocytoma Syndromes. In: Adam MP, Ardinger HH, Pagon RA, et al., eds. *GeneReviews®*. Seattle (WA): University of Washington, Seattle; 2008.

9. Favier J, Amar L, Gimenez-Roqueplo A-P. Paraganglioma and phaeochromocytoma: from genetics to personalized medicine. *Nat Rev Endocrinol*. 2015;11(2):101-111.
